# Enhancing a sustainable healthy working life: design of a clustered randomized controlled trial

**DOI:** 10.1186/1471-2458-10-461

**Published:** 2010-08-06

**Authors:** Wendy Koolhaas, Sandra Brouwer, Johan W Groothoff, Jac JL van der Klink

**Affiliations:** 1 Department of Health Sciences, Work and Health, University Medical Center Groningen, University of Groningen (FA10), Antonius Deusinglaan 1, 9713 AV Groningen, The Netherlands; 2 Graduate School for Health Research, University Medical Center Groningen, University of Groningen (FA10), Antonius Deusinglaan 1, 9713 AV Groningen, The Netherlands

## Abstract

**Background:**

To improve a sustainable healthy working life, we have developed the intervention 'Staying healthy at work', which endeavours to enhance work participation of employees aged 45 years and older by increasing their problem-solving capacity and stimulating their awareness of their role and responsibility towards a healthy working life. This research study aims to evaluate the process and the effectiveness of the intervention compared with care as usual.

**Methods/design:**

The study is a cluster-randomized controlled trial design (randomized at the supervisor level), with a 1-year follow-up. Workers aged 45 years and older have been enrolled in the study. Workers in the intervention group are receiving the intervention 'Staying healthy at work'. The main focus of the intervention is to promote a healthy working life of ageing workers by: (1) changing workers awareness and behaviour, by emphasizing their own decisive role in attaining goals; (2) improving the supervisors' ability to support workers in taking the necessary action, by means of enhancing knowledge and competence; and (3) enhancing the use of the human resource professionals and the occupational health tools available within the organization. The supervisors in the intervention group have been trained how to present themselves as a source of support for the worker. Workers in the control group are receiving care as usual; supervisors in the control group have not participated in the training. Measurements have been taken at baseline and will be followed up at 3, 6 and 12 months. The primary outcome measures are vitality, work ability and productivity. The secondary outcomes measures include fatigue, job strain, work attitude, self-efficacy and work engagement. A process evaluation will be conducted at both the supervisor and the worker levels, and satisfaction with the content of the intervention will be assessed.

**Discussion:**

The intervention 'Staying healthy at work' has the potential to provide evidence-based knowledge of an innovative method to promote a sustainable healthy working life in the older working population. The results of the study will be relevant for workers, employers, occupational health professionals and human resource professionals.

**Trial registration:**

The trial is registered with the Dutch Trial Register under number NTR2270.

## Background

Ageing of the workforce exerts pressure on society with respect to health, wealth and social insurance systems, which are inextricably linked [1]. Older workers are more vulnerable in the labour process, because of vitality and ageing problems affecting their daily performance and their ability to meet job competence requirements [2-6]. Ageing is associated with a higher sickness absenteeism rate [7], reduced work ability and decreased productivity [8-10]. Most societies are geared to retirement at around 65 years of age. An inability to meet work demands, due to ageing, forces older workers to leave the labour market before reaching retirement age [11,12]. In addition, the age of the working population is declining [13]. This situation affects government budgets and social securities, and puts pressure on the current arrangements for public pensions and healthcare [11,12].

The nature of work has changed over recent years due to globalization and information technologies (the 'new economy') in the Organisation for Economic Co-operation and Development (OECD) countries, and work-based life-long learning is required [14]. Moreover, the more dynamic market and shorter product cycles have resulted in fewer jobs, with frequent job changes over a working lifetime [14]. To keep workers in the labour market during the coming decades, employers should invest in education for and training of their employees, and the implementation of policies and working methods to enable workers to have the necessary competencies for longer working lives [12,15]. Due to these changes in the labour market, the employee-employer relationship has evolved from one of mutual loyalty to one based on personal gain. Workers are increasingly responsible for their own career, and they need to be aware of their own responsibility and decisive role in creating and/or maintaining a healthy work life. Most intervention studies to promote workers' health to extend working life, provide a lifestyle training programme, for a specific group of workers, to improve job retention [16], increase vitality [17] or decrease work disability and sickness absenteeism [18-21]. However, to gear the work demands and activities to the personal capabilities of the worker, in order to maintain and promote sustainable work participation, a strategy for older workers to solve problems with regard to ageing and chronic health conditions is required, involving good cooperation between the supervisor and the employee [22-24]. We have therefore developed an intervention to improve a healthy sustainable working life for workers aged 45 years and older.

Our intervention is called 'Staying healthy at work'. The goal of the intervention is to promote a healthy sustainable working life of older workers until retirement age by: (1) changing workers awareness and behaviour by emphasizing their own decisive role in attaining goals and giving them the feeling that they can be effective in carrying out the necessary actions; (2) improving supervisors' ability to support workers in taking the necessary actions by means of enhancing knowledge and competencies; and (3) enhancing the use of human resource professionals (HRPs) and occupational health tools available within the organization.

The rationale of the intervention is based on theoretical models and theories, on the results of a survey study of workers aged 45 years and older and on four expert meetings. A detailed description of the development of the intervention is presented in the Appendix (additional file 1). The primary aim of this intervention study is to evaluate the effectiveness of the 'Staying healthy at work' intervention compared with care as usual (CAU) on productivity, vitality and work ability. We hypothesized that after undergoing the intervention, older workers would improve sustainable work participation by using problem-solving strategies with regard to health problems or opportunities to create a healthy work situation compared with workers who received CAU. The secondary aims are to improve work attitude, self-efficacy and work engagement, and to decrease fatigue and job strain. A process evaluation will be conducted among workers and supervisors. To our knowledge, this is the first study focusing on a sustainable healthy working life.

## Methods/design

The methods and design of the intervention study are as described in the CONSORT statement and the extension for cluster-randomized trials [25,26].

**Figure 1:**
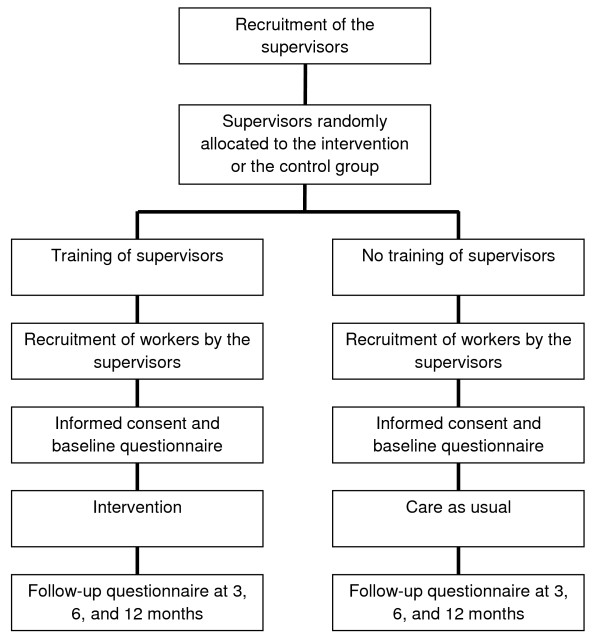
**Study design**. Overview of the study design of the intervention.

The study is designed as a two-armed cluster-randomized controlled trial (RCT) with a 1-year follow-up. In the study, the intervention group will be compared with CAU (Figure 1). Eligible workers could not be randomly assigned to supervisors in the intervention group or supervisors randomly assigned to eligible workers in the control group, because the supervisor and worker are bound to each other by their department. Moreover, training all supervisors before randomization was not possible because of the risk of data contamination. Therefore, cluster randomization was applied at the supervisor level [27]. Supervisors at the same department have been placed in either the intervention or the control group. Workers with their supervisor in the intervention group have been allocated to the intervention group, and workers with their supervisor in the control group have been allocated to the control group.

The Medical Ethical Board of the University Medical Center of Groningen informally approved the study design. They decided that this study did not need an ethical approval, because this study did not perform clinical research with medicinal product for human beings according to the Dutch law. Workers could participate voluntarily in this study, and they are free to leave the study at any time without further consequence. All workers signed an informed consent to participate in the study.

### Study population

Recruitment of participants took place at the University and the University Medical Center of Groningen. The source population consisted of workers aged 45 years and older from different departments: intensive care, administration, personnel and executive workers. Workers on long-term sick leave with no prospect of recovery, or workers who left the job within 1 year because of illness or pension have been excluded from the study.

#### Recruitment of the supervisors

Supervisors at the departments proposed by the HRPs were invited to participate in the study. Supervisors who were interested in the intervention method and were willing to participate in the study received information about the procedure.

### Procedure

The supervisor informed the workers of the upcoming intervention. Hereafter, the workers received a letter of invitation to participate in the study, describing the aim, content and set-up of the study. Workers were invited to return a signed informed consent to confirm their participation. Workers who were willing to participate in the study received a baseline questionnaire (paper version) and a postage-paid envelope.

### Intervention

#### Supervisor training in the intervention group

The supervisors received training before the implementation of the intervention, which consisted of two components. The first training of 2 hours focused on knowledge regarding a sustainable healthy working life, and on problem-solving techniques. After 2 weeks, the second training took place, which consisted of an active training module in which the problem-solving techniques were taught, and practiced by role play. In this 5-hour training module, the trainer was assisted by an actor. The supervisors were trained in skills to support the worker in basic problem-solving techniques. A structured method for identifying problems, solutions and applications of the solutions was offered, and skills to guide the worker in using this method were taught. Moreover, the supervisor was trained on how to present him- or herself as a source of support for the worker; not by taking over responsibilities, but by strengthening the autonomy of the worker. Furthermore, the supervisors received an overview of HRP and occupational health tools available within the organization, such as work adjustments, training and education. This should help them to advise their workers about which tools they could use to optimize work capacities and personal development, and thereby work participation.

#### Intervention for workers in the intervention group

##### Step 1: inventory of problems, solutions and degree of changeability

Workers in the intervention group received a booklet. The content of the booklet was based on the problem-solving strategy described by Fontana [28] and modified by van der Klink [29,30]. The booklet is designed by the researchers to help with clarifying and exploring problems with work functioning, working career and support needs, and stimulate workers to think about possible solutions. In the booklet, examples of problems at work due to ageing and support needs, possible solutions, and their role and responsibility in creating a sustainable working career are described. The workers made an inventory of experienced problems, barriers and support needs, including concrete examples of work situations in which these problems occur. In addition, workers could indicate career aspirations. Hereafter, workers determined the degree of changeability of each described point. Workers needed to consider which changes they could realize by themselves, either by changing the situation or by mobilizing support (e.g. supervisor, occupational physician, social worker or psychologist), or, when the situation could not be influenced, by learning how to cope with it, and accepting it. The booklet was sent to the supervisor after finishing these first steps.

##### Step 2: dialogue between worker and supervisor

The worker's inventory in the booklet was the input for the dialogue between worker and supervisor. The dialogue took place within 3 months of sending the booklet to the workers.

During the dialogue, the supervisor helped to establish the worker's experienced problems in work functioning, goals and solutions, on the process level. After identifying problems with work functioning, support needs and defining career opportunities, a concept action plan was made after brainstorming about the possibilities, actions and responsibilities of both the worker and his supervisor.

##### Step 3: Action plan

After the dialogue with the supervisor, the worker made an action plan for the next 1-year follow-up period. The booklet that workers received before the dialogue contained an example of an action plan format to help workers to create their own action plan. Within 2 weeks of the dialogue, the worker had to complete this action plan, including the solutions discussed with the supervisor. Workers are responsible for the execution of these actions during the following year - when necessary, with the support of the supervisor or other professionals. After 1 year, during the next annual assessment between worker and supervisor, the action plan will be evaluated and both results and process will be discussed.

#### Treatment of the control group

The supervisors in the control group did not receive training. If the intervention 'Staying healthy at work' proves to be effective, the supervisors in the control group will be trained in the future. Workers in the control group are being given CAU, which implies no structured support, but the possibility of counselling or support by HRPs or other professionals as required, or participating in training and human resource tools.

### Effect evaluation

To investigate the effectiveness of the intervention 'Staying healthy at work', all workers received a baseline questionnaire, and follow-up questionnaires are to be filled in at 3, 6 and 12 months.

#### Socio-demographic variables

At baseline, socio-demographic data (gender, age, level of education, nature of work, current work status, working hours a week) have been collected.

### Primary outcome

The primary outcomes of the study are: work ability, vitality and productivity.

#### Work ability

Work ability is being measured by the Work Ability Index (WAI) [31], a self-administered questionnaire comprising seven scales: (1) subjective estimation of current work ability compared with lifetime best (0-100 points); (2) subjective work ability in relation to both physical and mental demands of the work (2-10 points); (3) number of diagnosed diseases (1-7 points); (4) subjective estimation of work impairment due to diseases (1-6 points); (5) sickness absenteeism during the past year (1-5 points); (6) own prognosis of work ability after 2 years (1 or 4 of 7 points); and (7) psychological resources (enjoyment of daily tasks, activity and life spirit, optimism about the future) (1-4 points). The final WAI score is calculated by summation of all scale scores and can range from 7 to 49 points. The reliability and validity of the WAI are acceptable [32,33]. Higher scores on the WAI indicate a better work ability. Based on this WAI score, the individual's work ability can be classified into four categories: poor (7-27 points); moderate (28-36 points); good (37-43 points); and excellent (44-49 points).

#### Vitality

Improvements in health-related outcomes are being evaluated with the self-reported 12-Item Short Form Health Survey (SF-12), an abbreviated version of the 36-Item Short Form Health Survey [34-36]. The SF-12 provides two summary scores, the Physical Component Summary (PCS) score, which represents what a person can do, and the Mental Component Summary (MCS) score, which represents how a person feels. The mean PCS and MCS of the general population are 50, with a standard deviation of 10. A higher score means a better quality of life.

#### Productivity

Productivity is being measured with the QQ method, which aims at measuring the quantity and quality of work performed on a daily basis [37]. The workers indicate how much work they actually perform during regular hours on their last regular work day during a working week as compared with a normal week day. The quantity of productivity is measured on a 10-point rating scale with 0 representing 'nothing' and 10 representing 'normal quantity'. Meerdings et al. [9] showed that self-reported productivity in the QQ measurement correlated significantly with objective work output.

### Secondary outcome

The secondary outcomes of the study are changes in fatigue, psychosocial work characteristics, work attitude, self-efficacy and work engagement.

#### Fatigue

To determine the level of fatigue, we are using the Checklist Individual Strength (CIS) [38]. The CIS is a 20-item well-evaluated questionnaire for the working population, measuring four aspects of fatigue in separate scales. In this study, the subscale of the subjective feeling of fatigue (eight items) is being used. The items are scored on 7-point Likert scales ranging from 'Yes, that is true' to 'No, that is not true'. Higher scores on the separate scales indicate higher degrees of fatigue [38].

#### Psychosocial work characteristics

The Job Content Questionnaire (JCQ) is a self-administrated questionnaire designed to measure job strain [39]. The domains assessed are job demands (5 items), decision authority (3 items), skill discretion (6 items), social support from supervisors (4 items) and co-worker support (4 items). Each domain is rated on a 4-point scale from 'strongly disagree' (most negative) to 'strongly agree' (most positive). The reliability of the scales is good [40].

#### Work attitude

Perceived work attitude is being measured with a Dutch language version of the Work Involvement Scale (WIS-DLV), reflecting the degree to which a person wants to be engaged in work [41]. The questionnaire consists of six items; with responses on a 1-4 point scale (strongly disagree, disagree, agree, strongly agree). Higher scores on the WIS-DLV indicate more positive attitude towards work.

#### Self-efficacy

Self-efficacy is being measured with the standardized Dutch version of the General Self-Efficacy Scale [42], assessing the subjects' expectations of their general capacities [43]. This 16-item questionnaire incorporates three subscales: willingness to exert effort in completing the behaviour, persistence in the face of adversity, and willingness to initiate behaviour. It consists of five response items (ranging from disagree to agree); higher scores indicate a higher self-efficacy.

#### Work engagement

Work engagement is being measured by a short Dutch version of the Utrecht Work Engagement Scale (UWES-9), which enquires how often the respondents currently experience positive emotions at work [44]. The UWES-9 consists of nine items rated on a 7-point scale ranging from 'never' (0) to 'always/every day' (6). The items are divided into the subscales vigour, dedication and absorption. A total score is obtained by averaging the individual item scores (possible range 0-6). The internal reliability and validity of the Dutch UWES-9 are acceptable [45].

### Process evaluation

The process evaluation will examine the applicability to implement the intervention 'Staying healthy at work' at both the supervisor and the worker level. The supervisors in the intervention group received a questionnaire before and will receive one after the training to examine the quality of the training, the content of the training, and the added value of the training, as a strategy to improve the communication with the workers regarding work participation and work functioning.

Among the workers of the intervention group, experiences with the use of the intervention will be evaluated in the follow-up questionnaires. Workers were asked to evaluate the content and the relevance of the information leaflets and the booklet to prepare for the dialogue. Also the dialogue with the supervisor will be evaluated in the questionnaire after 3 months. All follow-up questionnaires contain items about the support of the supervisor, the contribution of the booklet to help make an inventory and action plan, and the workers' experiences with the problem-solving strategy to stimulate communication about work performance. In addition, questions will be asked about the enhanced problem-solving capacity and awareness with regard to the workers' own role and responsibility towards a healthy and motivating work situation. Workers will also be asked to evaluate whether the work-related situations they wanted to improve have actually improved, whether they had used HR tools, training, education or contacted professionals during the intervention and if they had attained the goal they had in mind at the start of the intervention. Both workers and supervisors received questions at the baseline and will receive follow-up questionnaires about the readiness for change concerning the intervention.

Reasons for complying or not complying will be asked in the follow-up workers' questionnaire as well, in order to gain insight into the potential success of implementation.

### Statistical analyses

All analyses will be performed according to the intention-to-treat principle. Baseline characteristics of workers will be analyzed for differences between the intervention and the control groups. Differences between the intervention and the control groups in changes on the outcome variables will be performed with multi-level longitudinal analysis. Intra-cluster correlations will be determined for all primary outcome variables. Effects of the intervention will be controlled with covariates, such as gender and job type. Further analyses will include the comparison of the secondary outcomes at follow-up between the two arms. For all analyses a two-tailed significance level of p < 0.05 is considered to be statistically significant. All analyses will be carried out with the statistical package SPSS version 16.0 (SPSS Inc. Chicago, IL, USA).

### Power calculations and sample size

Work ability, measured with the Work Ability Index (WAI) [46] is the primary outcome measure for the power calculation. The range of the summative index of the WAI is 7-49, which is classified into four subgroups: poor (7-27), moderate (28-36), good (37-43), and excellent (44-49) [31]. The target of this intervention study is to increase the mean scores of the workers to those of the next subgroup. This implies a minimum increase of 5.4 (24%) for workers with a poor work ability, and minimum increase of 3.7 (11% and 9%, respectively) for workers with a moderate and good work ability before the intervention. For the workers with an excellent WAI score at baseline, the aim is to maintain the score. The survey study demonstrates that the effect size of workers is high (≥0.80).

We used Optimal Design [47,48] to calculate the optimal sample size by a power of 0.80 for testing the treatment effect in this cluster-randomized trial with an intervention and a control group in a repeated measurement design. A literature search on the role of the supervisor to the worker's attitude [24,49] showed an intra-class correlation (ICC) between 0.11 and 0.26 for supervisors. We used the most extreme value (ICC = 0.26) for the power calculation. A total of 20 supervisors are enough to reach a power of 80%.

Calculating from 80% power (two-sided, alpha = 0.05, 20 supervisors) a sample size of eight workers per supervisor are needed to determine a significant difference between the intervention and the control groups. The eight workers must be viewed as the average number of workers per supervisor. This requires at least 80 workers in each group. Allowing for a loss to follow-up of 15%, a sample size of 92 workers (in each group) is required.

### Blinding

The validity of the study will be compromised if information is known about the intervention in the control group. To minimize data contamination, pre-randomization at the supervisor level is applied for allocating the workers into the intervention or the control group. Therefore, different information about the study can be provided to workers in the intervention and the control groups [27]. Workers in the control group do not know the content and design of the intervention 'Staying healthy at work'. Whereas workers in the control group are blinded to the intervention, blinding of the supervisors was not possible. Supervisors in the control and the intervention groups have been informed about the content of the intervention, because they already knew the set-up of the study prior to their decision to participate.

## Discussion

The results of this cluster-randomized controlled study will provide input for an evidence-based intervention which may improve a sustainable healthy working life of workers aged 45 years and older. The intervention offers a structured method for workers to communicate with their supervisor about their work environment, barriers to work performance and career opportunities. Aspects concerning intervention procedure are described in the protocol; however, supervisors and workers are free to choose specific tools at each phase of the process. We assume that such a structured (non-protocol) intervention strategy giving workers the opportunity to make an action plan for the next 1-year follow-up period, will be an effective way to create a sustainable, healthy, working life for older workers. Moreover, this method has been shown to be effective in shortening sick-leave duration by workers on their first sick leave [50].

### Strengths

One of the main strengths of the intervention 'Staying healthy at work' is that it offers a strategy to improve the problem-solving capacity of both workers and supervisors. The workers will be trained to be aware of their own decisive role in attaining goals, and they will learn how to cope with future problems related to work participation. The skills of the supervisors will be improved by strengthening the workers' ability to act and make decisions autonomously, and to not take over the workers' responsibilities. Additional value of the intervention is that the method will contribute to the annual assessment, between supervisor and worker, of work functioning and participation. The intervention method can be incorporated into the annual worker assessments within the organization. Another strength of the intervention is the close cooperation with the HRPs during the development of the intervention. This offers a tool and a process that fit in with the existing company policy and improves the likelihood of effectiveness of the intervention. Furthermore, the intervention will contribute to the knowledge and the use of the current tools, education and training within the organization to enhance work participation. Although many organizations have these tools available in their current policy, supervisors and workers are often unaware of the opportunities they have aimed at improving the working environment. The fact that the perspectives of the workers on work functioning will be taken into account is also a strength of this study. We presume that this will lead to a better compliance of the workers to participate actively in the intervention programme. This will improve the effectiveness of the study. Finally, the cluster design of the study reduces the risk of data contamination between workers in the intervention and the control groups. Supervisors are randomly assigned to the intervention or the control group. Pre-randomization enables workers in the intervention group to be informed separately, and therefore makes it possible to blind the workers in the control group to the study condition.

### Weaknesses

The variations between the supervisors is a drawback of the design. Supervisors in the intervention group are trained to apply the problem-solving capacity strategy and to present themselves as a source of support to the workers. Variation between supervisors, such as differences in knowledge, capabilities, experience with communication, personality and readiness for change, can influence the results of this study, because of the decisive role of the supervisor during the dialogue between worker and supervisor. The process evaluation of the current study will give insight into the role of the supervisor during the dialogue and the follow-up of the study. Furthermore, a longer follow-up than the 12 months, as planned in the design, is preferable to investigate longer term intervention effects on a sustainable healthy working life. Workers make an action plan for the coming year based on one or two points of the inventory of problems, support needs and working career in the booklet. One year later, during the next dialogue between worker and supervisor, the actions will be evaluated and both results and the process will be discussed. In addition, they make an agreement about the next action to optimize work performance. Therefore, it would be advisable to follow the workers for more than 12 months. Another limitation of our study is that the evaluation of effect consists of measuring enhanced work ability, vitality and productivity. Moreover, no economical cost-effectiveness evaluations are included.

### Relevance of the study

To pro-actively address the issue of a shifting workforce composition, companies must anticipate and identify workforce issues within their organizations, and also develop strategies to effectively mitigate any workforce-related risks. A healthy working life requires close attention being paid to each workers' life-cycle phase (learning, applying, providing and diminishing) and individual demographic characteristics, such as age and health. A policy to provide a diverse, challenging and balanced working life is needed to increase knowledge, motivation and thereby participation. This study is focusing on enhancing the problem-solving capacity of workers to enhance work participation. The intervention 'Staying healthy at work' is not only designed to enhance work participation, but also to enhance the workers' resources and capabilities to continue working in good health until retirement.

## Abbreviations

HRP(s): human recourse (management); CAU: care as usual; WAI: work ability index; PCS: physical component summary; MCS: mental component summary; QQ: quantity and quality; CIS: checklist individual strength; JCQ: job content questionnaire; WIS: work involvement scale; UWES: Utrecht work engagement scale; ICC: intraclass correlation.

## Competing interests

The authors declare that they have no competing interests.

## Authors' contributions

WK, SB, JWG en JvdK designed the study. WK and JvdK developed the intervention. WK and SB wrote the study protocol which was discussed by all authors leading to the final design. WK wrote the final manuscript, which was discussed, edited and revised by all authors. All authors read and approved the final manuscript.

## Pre-publication history

The pre-publication history for this paper can be accessed here:

http://www.biomedcentral.com/1471-2458/10/461/prepub

## Supplementary Material

Additional file 1 **Development of the intervention**. Specification of the development of the intervention [51-64] Click here for file
