# Supplementary material for: Enhancing a sustainable healthy working life: design of a clustered randomized controlled trial
Source: BMC Public Health. 2010 Aug 6;10:461. doi: 10.1186/1471-2458-10-461 (PMC2928202; doi:10.1186/1471-2458-10-461)
Supplement: Additional file 1 — Development of the intervention. Specification of the development of the intervention [51-64] [file 1471-2458-10-461-S1.DOC]

**Additional files**

**Additional file 1**

Development of the intervention

To determine the content of the intervention, the following steps were taken: literature search, survey study and consultant meetings. Based on this information, we defined the three objectives of the intervention.

**Literature search**

The rationale of the intervention is based on a number of theories and models: problem-solving, empowerment and behavioural change models.

The problem-solving strategy is described in the guideline ‘Management of mental health problems of workers by occupational physicians’ of the Netherlands Society of Occupational Medicine [29,30]. The goal of the guideline is to support workers to control their own functioning in the work situation. A structured strategy for identifying problems, solutions and applications of solutions offers the workers the tools to increase their problem-solving capacity. This strategy helps them to find out that they can always influence the impact of a situation by: (1) changing the situation by themselves; (2) mobilizing the support of others; or (3) accepting the situation [30]. It contributes to the workers’ belief that they are capable of performing in a certain manner to attain goals, and thereby strengthens the their self-efficacy [51,52]. The guideline has been shown to be effective in shortening the sick leave duration of employees who are on long-term sickness absence because of mental health disorders [50,53].

The empowerment philosophy is based on the premise that human beings have the capacity to make choices themselves and are responsible for the consequences of their choices [54]. Varekamp (2009) and Crawford Shearer (2010) [55,56]show that empowerment-based interventions improve health, with purposeful participation in goal attainment and well being. Empowered workers are able to instigate changes in their personal behaviour and social situation, and also to influence the organization environment they work for. [54].

Behavioural change models are frequently used in the development and implementation of health-promoting interventions [57-60]. An example of a determinant model that has been applied to various types of health-related behaviour is the Attitude – Social influence – self-Efficacy (ASE) model derived from the theory of planned behaviour [51] and the social learning theory [52]. The ASE model is based on the assumption that the intention to engage in behaviour is the result of goal attitude (the positive and negative evaluation of the expected outcome of a certain behaviour), subjective norms (the belief about what others think of the behaviour, as derived from the behaviour and/or direct feedback of significant others), and self-efficacy (the confidence in being able to carry out a set of specified activities) towards performing the specific behaviour. The influence of the ASE model on the intention to change behaviour has been examined in several studies. Research shows that the ASE model predicts changes in injury-preventing behaviour [58], eating behaviour [60], changes in physical activity [57,59] and the intention to return to work after sickness leave [61]. Obviously, an ASE-based intervention is aimed at changing the determinants for intention into behaviour and to remove the barriers to performing the desirable behaviour.

Finally, the International Classification of Functioning, disability and health (ICF) has been a useful framework to classify and interpret relevant aspects with regard to disability and participation [62,63].

**Survey of the problems, barriers, facilitators and support needs due to ageing**

A survey study has been conducted to gather information about the problems experienced in work performance due to ageing, barriers and facilitators in the work situation and support needs to continue throughout the working life of workers aged 45 years and older [64]. Problems due to ageing, with regard to a sustainable working life, were reported by 41% of the workers; 37% reported a chronic health condition. Barriers to performing work tasks were reported by 59% of the workers who experienced problems due to ageing, and to continue working life in the coming years, 68% of the workers reported support needs. Most frequently reported barriers to work participation are physical problems (muscle function decline), cognitive problems (concentration lapses and memory deterioration) and mental problems (energy-driven functions based on psychological and physiological mechanisms) due to ageing. The most frequently experienced barriers were concentration, work-pace and mobility; facilitators are support from colleagues and supervisors, and opportunities for personal development. The results show that factors to enhance the working life of workers aged 45 years and older are found at the personal and the organizational levels. These findings emphasize the need for a joint – organization and worker – approach.

**Consultant meeting group**

Based on the literature search and survey study, a concept of the intervention has been developed and discussed during consultant meetings with experts in the field of work and health. First the content and practicability of the intervention were discussed in one session with two senior researchers (from national institutes with experience of occupational health intervention programmes) and two practising occupational health physicians. The importance of informing workers about the research as well as the content and organization of the study were discussed during the meeting. After revision of the recruitment procedure and the outcomes to define a sustainable working life, a meeting with the head of the human resources department of the participating organization took place. During this meeting, the importance of paying attention to the ageing workforce was presented, especially the benefits of sustainable healthy workers and the possibility of integrating the intervention into the current human resources system. This meeting led to some restrictions on the implementation of the intervention, namely the number of participants, the preference of participating departments, caused by the high percentage of older workers, and the selection on the suitability of the department, based on the current atmosphere. In addition, departments undergoing other types of intervention studies were excluded from our intervention study. The HRPs recommend measuring and supporting the use of current tools, education and training, to enhance work adjustments and personal development, because these tools are currently underused by workers.

After the second revision of the intervention, managers and supervisors at the proposed participating organizations were informed about the content of the intervention and the research project by their HRP. The managers and supervisors at the eligible departments, i.e. departments with the highest percentage of workers aged 45 years and older, were recruited by their HRPs. The researchers sent an information leaflet by post to these managers and supervisors, and consulted with all departments interested in participating in the study about the content of the intervention. Some parts of the concept intervention were omitted due to lack of support of the managers or supervisors, i.e. participation of all ages and a 2-day training of the supervisor.
